# Peripheral Nerve Protection Strategies: Recent Advances and Potential Clinical Applications

**DOI:** 10.3390/jfb16050153

**Published:** 2025-04-24

**Authors:** Weronika Radecka, Wiktoria Nogalska, Maria Siemionow

**Affiliations:** 1 Department of Orthopaedics, University of Illinois at Chicago, Chicago, IL 60607, USA; radecka.weronika@gmail.com (W.R.); nogalskawiktoria@gmail.com (W.N.); 2 Department of Anatomy, Institute of Medical Sciences, University of Opole, 45040 Opole, Poland

**Keywords:** nerve protection, nerve protector, nerve regeneration, nerve injuries, regenerative medicine, nerve repair, nerve wrap, synthetic nerve wrap, biological nerve wrap, tissue engineering

## Abstract

Peripheral nerve injuries (PNIs) are a significant clinical challenge, often resulting in persistent sensory and motor deficits despite surgical repair. Autologous nerve grafts remain the gold standard for repair; however, outcomes are frequently suboptimal due to donor site morbidity and inconsistent functional recovery. A major obstacle in nerve regeneration is the formation of postoperative adhesions and fibrosis, which impede healing and necessitate revision surgeries. Nerve protectors from biological, synthetic, and hybrid materials offer a promising tissue engineering strategy to enhance nerve regeneration. These protectors are applied as a protective barrier when a nerve is severed without the gap, allowing for direct repair. They provide mechanical support and reduce scarring. Biocompatible biological wraps, including vascularized fat flaps, vein wraps, collagen-based materials, human amniotic membrane (hAM), porcine small intestinal submucosa (PSIS), and chitosan, modulate immune responses and promote vascularization. Synthetic alternatives, like polycaprolactone (PCL), provide mechanical stability with controlled degradation. Hybrid wraps, such as PCL-amnion, combine the benefits of both. Despite optimistic results, the heterogeneity of study methodologies hinders direct comparisons and standardization. This review highlights the latest developments in nerve wraps, their clinical applications, limitations, and future potential, guiding clinicians in selecting the most appropriate materials for peripheral nerve repair.

## 1. Introduction

Peripheral nerve injuries (PNIs) represent a significant clinical challenge affecting millions worldwide, often resulting in long-term disability, including chronic neuropathic pain, sensory loss, and motor deficits [1]. Effective management of PNI is essential for the restoration of function and improved quality of life of the affected patients [2]. However, despite advancements in surgical techniques and the introduction of biomaterials for support of nerve regeneration, managing PNI still remains complex, with patient recovery outcomes varying significantly [3].

Historically, the understanding of PNI was limited; by the late 18th century, it was widely believed that regeneration of damaged peripheral nerves was impossible [4]. Modern microsurgical techniques improve nerve regeneration after trauma; however, still, only half of the patients report satisfactory recovery, whereas one-third of patients may see little or no improvement [5]. Various nerve repair techniques offer distinct advantages and potential complications. This highlights the ongoing need for innovation and optimization in therapeutic strategies for nerve injuries [6].

Scar tissue formation is a common consequence of PNI and surgical interventions, significantly impeding nerve regeneration and predisposing patients to complications such as traction neuropathy. Understanding the mechanisms behind scar formation is crucial for improving treatment outcomes and advancing regenerative strategies. Effective prevention of scar tissue formation remains a key focus in the development of biomaterials and surgical techniques [2].

**Figure 1:**
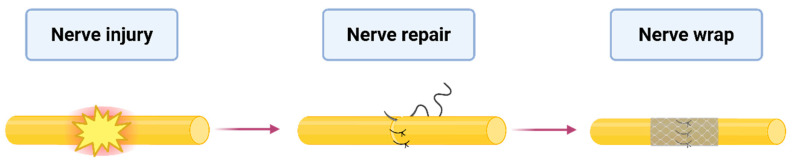
Application of the nerve protector. The figure illustrates the application of nerve-protecting wrap after nerve injury and repair.

One of the most promising innovations in this field is the use of a nerve wrap—a bioengineered barrier that surrounds the nerve, providing a protective interface [7]. Nerve wraps are used when a nerve is transected without a resulting gap, where direct repair is feasible, and grafting is not required (Figure 1). An ideal nerve protector should be biocompatible, minimize the risk of fibrosis and scarring, and ensure sufficient nerve mobility [8,9]. The concept of nerve wrapping dates back to 1989, when Masear first proposed using an autologous vein as a protective sheath [10]. Since then, a diverse array of biological, synthetic, and hybrid nerve protectors has been introduced, each designed to support the natural regeneration process [9].

**Figure 2:**
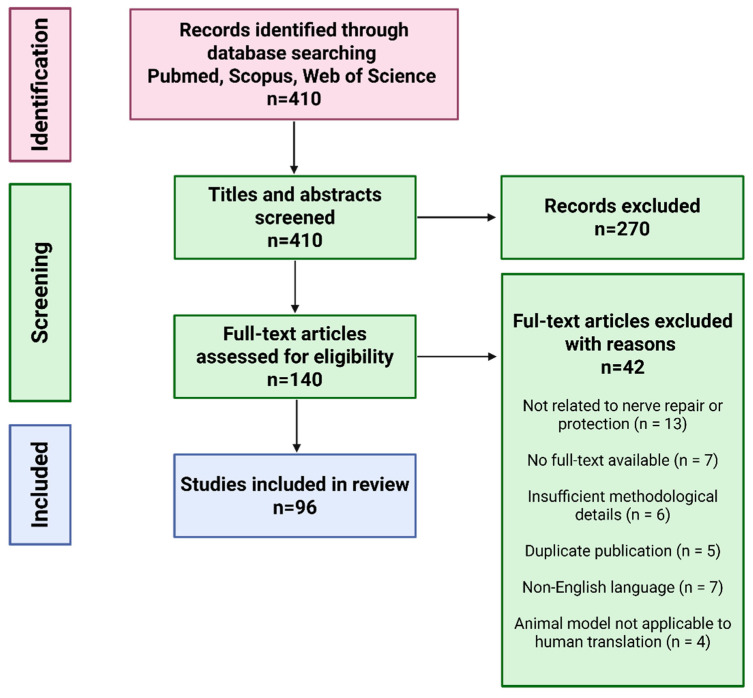
Flow chart of article preselection, selection, and exclusion for nerve wrapping materials review.

This article presents a narrative review of current strategies and materials used to protect peripheral nerves from scar tissue formation, fibrosis, and inflammation following nerve injuries or surgical procedures. Relevant literature was identified through structured searches of PubMed, Scopus, and Web of Science using keyword combinations such as “peripheral nerve protection,” “nerve protector,” “nerve wrap,” “nerve injury,” “nerve regeneration,” and “regenerative medicine” (Figure 2). Original experimental and clinical studies published in English were included, with priority given to articles from 2020 to 2025 to ensure coverage of the most recent advancements in the field. Additional studies were identified by screening the reference lists of the selected articles. The review is strictly focused on peripheral nerve protection; studies addressing the central nervous system or biomaterials developed exclusively for other tissue types were excluded.

This review provides a comprehensive analysis of the current progress in PNI management, emphasizing cutting-edge surgical techniques and biomaterials. It evaluates their efficacy, limitations, and clinical potential while highlighting their impact on patient outcomes. Additionally, it identifies remaining challenges and suggests future research directions for peripheral nerve repair.

## 2. Nerve Protection Strategies: Biological vs. Synthetic Nerve Wraps/Protectors

**Figure 3:**
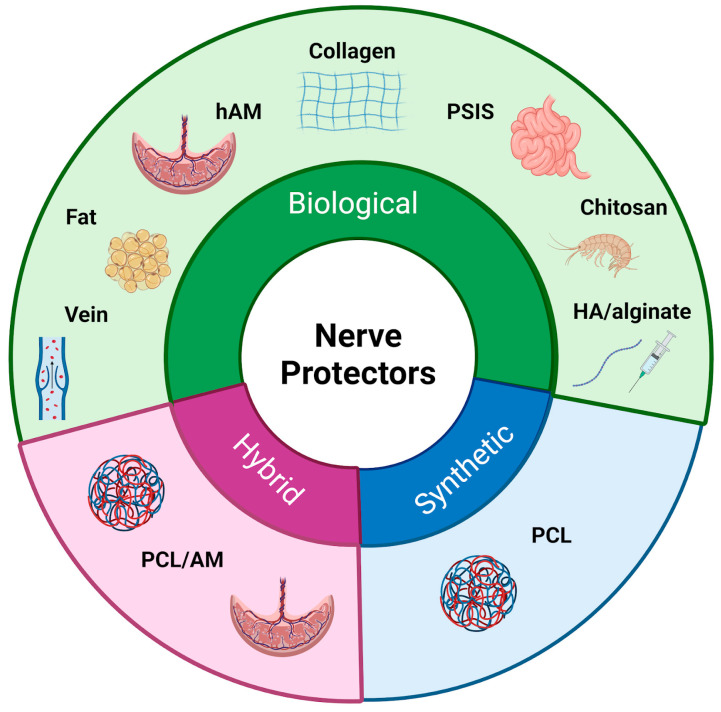
Classification of nerve protectors. The figure presents a classification of nerve protectors into three main types: biological, synthetic, and hybrid materials.

Following the initial exploration of autologous tissues for nerve protection, research has expanded to include other biological materials with regenerative potential [11] (Figure 3) (Table 1). Recognizing that natural tissues might provide an optimal environment for nerve regeneration, researchers have developed biological nerve wraps. By integrating with the surrounding tissue and utilizing intrinsic repair mechanisms, these wraps promote axonal regrowth and regeneration. As the complexity of nerve injuries became more evident, the focus shifted from tissue derivatives to engineered scaffolds, resulting in the development of biological and synthetic nerve wraps [5,12,13]. Despite differences in composition and degradation profiles, both types of materials function as physical barriers, shielding regenerating nerves from external compression and scar tissue formation [11]. The choice between biological versus synthetic wraps depends on various factors, including clinical indications, extent of injury, anatomical location, patient-specific considerations, and surgeon preference [14].

## 3. Biological Nerve Protectors

Pursuing effective nerve repair strategies has long driven advancements in regenerative medicine, with biological nerve protectors emerging as a cornerstone in this field. Derived from human or animal tissue, they share several key characteristics that contribute to their effectiveness. Their inherent biocompatibility significantly reduces the risk of immune rejection and enhances the likelihood of successful integration into the body [15]. Additionally, their resorbable nature ensures they naturally degrade over time, eliminating the need for surgical removal [16]. Many biological protectors, such as autologous fat flaps and vein wraps, facilitate nerve gliding and reduce adhesions. Others, like collagen-based wraps and human amniotic membrane (hAM), contribute to vascularization and immune modulation, further enhancing nerve recovery. Their anti-inflammatory and antifibrotic properties mitigate perineural scarring. However, they come with certain disadvantages, such as rapid degradation, which may lead to premature loss of structural support before the nerve has fully regenerated [17]. Seven nerve-protecting wraps made of porcine submucosa, collagen, and calcium alginate have received FDA approval for clinical use in the United States [18,19] (Table 2).

### 3.1. Vein Wraps for Enhancement of Nerve Regeneration

Since Masear first introduced vein wrapping (VW) for nerve protection, autologous veins have been utilized to support nerve repair techniques [10] (Table 3). This method employs freshly harvested vein tissue as a protective barrier around nerves, using the vein’s smooth intimal layer to create a gliding surface [20]. VW is accessible and cost effective, making it suitable for addressing both traumatic nerve injuries and chronic compressive neuropathies [21]. Studies suggest that VW may contribute to neuroprotection by promoting the release of basic fibroblast growth factor (bFGF), which triggers the induction of heme oxygenase-1 (HO-1), an enzyme with immunomodulatory and antinociceptive effects. In rat sciatic nerve injury models, this mechanism has shown promise in managing pain [22,23]. Furthermore, vein-derived cytokines such as interleukin-4 (IL-4) and interleukin-10 (IL-10) enhance the expression of M2 macrophage markers like CD206 and Arginase-1 (ARG1), contributing to an anti-inflammatory environment and facilitating tissue regeneration while reducing neuropathic pain [24]. Clinically, VW has been shown to be an effective technique for preventing complications like perineural scarring and neuromas. Application of VW following nerve repair demonstrated improved motor and sensory recovery compared to standard neurorrhaphy [20]. However, the clinical application of VW can present challenges such as donor site morbidity, longer surgery time, and technical difficulties, which must be carefully weighed when considering the application of VW for enhancement of peripheral nerve regeneration [8].

### 3.2. Vascularized Fat Flaps for Nerve Protection

Different materials for nerve protection have emerged over the last few decades. Both vascularized and free-fat flaps have been used for peripheral nerve protection. However, the vascularized pedicle flaps have become more popular in recent years. These flaps offer several advantages, such as revascularizing nerve tissue, thereby reducing fibrosis, preventing nerve adherence to surrounding structures, and allowing nerve gliding within its natural surroundings [32]. They have gained attention due to their easy availability and abundance. Adipose tissue is rich in adipose-derived stem cells (ADSCs), which can differentiate into various cell types, particularly Schwann-like cells, and guide regenerating axons [33,34,35]. The rich capillary network within adipose tissue helps sustain Schwann cells and other supporting cellular components, ensuring a steady influx of growth factors. The procedure is minimally invasive, safe, and effective in ameliorating persistent neuropathic pain and promoting nerve recovery, particularly in cases of chronic nerve injury or recalcitrant carpal tunnel syndrome [36]. As a result, there is a reduced need for antineuropathic medications, thus enhancing patients’ quality of life while minimizing drug side effects [37]. Clinical studies have shown that the application of a vascularized fat flap during the anterior subcutaneous transposition of the ulnar nerve reduces perineural scarring and supports nerve regeneration by mimicking the natural fatty surroundings of peripheral nerves [38]. However, despite many advantages, there are still challenges, such as donor site morbidity and extended operative time due to the fat flap harvest, which must be considered when choosing fat flaps as the protective barrier against scar and adhesion formation [39].

### 3.3. Human Amniotic Membrane as the Protective Barrier

The human amniotic membrane (hAM), well recognized for its wound-healing properties, has emerged as an effective biomaterial for the enhancement of peripheral nerve regeneration. Derived from the placenta, this non-immunogenic tissue is rich in collagen, growth factors, and glycoproteins [40]. Its extracellular matrix is designed to promote wound healing and modulate cellular processes. hAM’s regenerative potential is further enhanced by its anti-inflammatory cytokines, along with its antifibrotic and antibacterial properties that facilitate nerve healing and reduce the incidence of postoperative adhesions [41,42]. One of the key advantages of hAM is its lack of human leukocyte antigen (HLA) expression, significantly reducing the risk of immune rejection and enabling broad clinical applicability without complications typically associated with foreign tissue materials [43]. In a rat model of complete common peroneal nerve transection, the effect of amniotic membrane (AM) wrapping was evaluated by comparing two groups: the suture group (nerve transection with end-to-end repair) and the AM wrap group (nerve transection with end-to-end repair followed by two-layer AM wrapping). AM application significantly enhanced functional recovery, as evidenced by improved nerve conduction velocity and axonal transport. In contrast, the suture group without wrapping showed limited muscle regeneration and persistent perineural fibrosis [44]. Similar benefits were observed in clinical settings, where patients undergoing cubital tunnel decompression supported by wrapping the ulnar nerve with hAM experienced a reduced recurrence of paresthesias compared to those without hAM application [45]. The widespread availability, cost-effectiveness, and potent biological activity of hAM make it a highly advantageous tool in neuroprotection strategies. As research continues to support its clinical applications, hAM is positioned as a leading choice for enhancing nerve recovery and reducing post-surgical complications following PNI [46].

### 3.4. Bioresorbable Collagen for Nerve Regeneration

Once regarded as a simple structural protein, collagen has now become one of the key materials used to support peripheral nerve repair due to its biocompatibility and low immunogenicity [47]. As a natural component of the extracellular matrix (ECM), collagen provides a conducive scaffold for cellular attachment, Schwann cell adhesion, proliferation, and migration, supporting the alignment of regenerating axons [48]. Unlike synthetic materials, which may provoke chronic foreign body reactions, purified collagen exhibits minimal antigenicity, particularly when enzymatically treated to remove immunogenic telopeptides or chemically crosslinked to modulate degradation kinetics [49]. The bioresorbable nature of collagen ensures gradual degradation, reducing the risks associated with foreign body materials and allowing for controlled healing without the need for surgical removal [50,51]. Preclinical studies using rat sciatic nerve models confirmed that the selective permeability of the collagen Type 1 wrap allowed the diffusion of essential micronutrients and growth factors while preventing infiltration by fibroblasts [52]. Clinically, collagen nerve wraps are effective in preserving nerve tissue homeostasis in conditions such as carpal and cubital tunnel syndrome [53,54]. Commercially distributed, FDA-approved products, such as NeuraWrap™ (Integra LifeSciences Corporation, Plainsboro, NJ, USA) and NeuroMend™ Wrap (Stryker Corp., Kalamazoo, MI, USA), are designed to unroll and naturally conform to the shape of the injured nerve, ensuring an optimal fit without compression [55,56]. NeuraWrap™ and NeuroMend™ mainly differ in their resorption rates, with NeuraWrap™ breaking down over 36 to 48 months, while NeuroMend™ degrades within 4 to 8 months [57]. Despite their advantages, the differences in resorption rates, influenced by crosslinking and host enzymatic activity factors, may lead to nerve compression [58]. Furthermore, while inflammatory responses are generally mild, occasional foreign body reactions have been reported, necessitating careful material selection and post-implantation monitoring [59].

### 3.5. Porcine-Derived Biomaterials for Nerve Regeneration

In the complex landscape of nerve repair, where meticulous surgical technique and biocompatibility of the applied materials dictate the success of regeneration, porcine small intestine submucosa (PSIS) has emerged as a compelling biomaterial. With its structural resemblance to the native human extracellular matrix (ECM), PSIS provides a supportive microenvironment for nerve healing. In addition, it fully degrades within 3 months following application [60,61]. Advances in decellularization techniques have optimized the safety profile of these matrices by reducing immunogenicity while preserving their bioactive components, such as collagen (Types 1 and 3), laminin, and fibronectin, key factors in axonal growth, Schwann cell migration, and myelination [62]. Pre-clinical studies have demonstrated encouraging results, including revascularization and remodeling into connective tissue similar to the nerve epineurium after implantation [28]. Clinically, PSIS wraps have gained attention, particularly in recurrent and persistent carpal tunnel syndrome and cubital tunnel syndrome, where chronic nerve compression is a major concern [63,64]. Their application in upper extremity surgeries has been associated with reduced pain and improved functional outcomes [65]. Investigations into PSIS use in ulnar nerve revisions and other peripheral nerve conditions highlight its ability to reduce inflammatory reactions [66]. This anti-inflammatory effect has been attributed to the material’s ability to modulate the local immune response, including the downregulation of pro-inflammatory cytokines, such as TNF-α and IL-1β, and the promotion of a macrophage phenotypic shift from the pro-inflammatory M1 to the pro-regenerative M2 subtype, thereby creating a microenvironment favorable for nerve healing and axonal regeneration [67]. In the commercial space, three variants of PSIS nerve repair solutions are available, including Axoguard^®^ Nerve Protector (Axogen, Inc. Alachua, FL, USA), the more advanced Axoguard^®^ HA+ Nerve Protector (Axogen, Inc. Alachua, FL, USA), which incorporates a resorbable hyaluronate–alginate gel layer, and Nerve Tape^®^ (BioCircuit Technologies, Inc, Atlanta, GA, USA), a suture-less coaptation device [68]. Nerve Tape utilizes microhooks embedded in a flexible, biocompatible material to enable quick and precise alignment of nerve ends without sutures, providing an effective alternative to traditional microsuture neurorrhaphy [69,70]. The presented innovations reflect a paradigm shift in peripheral nerve repair techniques, where the application of biomaterials bridges the gap between surgical intervention and natural regeneration. While PSIS-based products are generally well tolerated, rare immune responses have been reported and should be taken into consideration when making choices between different biomaterials used for the enhancement of nerve regeneration [71].

### 3.6. Chitosan-Based Nerve Protectors

As the quest for innovative biomaterials in nerve repair continues, chitosan nerve sheets emerge as an alternative in regenerative medicine. Derived from chitin, a natural polysaccharide from the exoskeletons of crustaceans, chitosan is a biodegradable and biocompatible material [72]. It is rich in deacetylated chitin and has shown potential in inhibiting extraneural scarring and promoting neural regeneration, as demonstrated in experimental studies on rat tibial nerves [73]. Research suggests it effectively supports nerve regeneration in sheets and as a drug carrier, increasing its clinical potential. In particular, its ability to reduce scarring and support Schwann cell migration indicates its possible use as an alternative to currently used approaches [74]. Chitosan’s inherent antimicrobial properties further reduce the risk of infection [75,76]. However, the degradation time of chitosan-based wraps can vary depending on factors such as the degree of acetylation and the specific formulation used. Some studies have reported that chitosan nerve wraps did not show significant degradation after extended periods, suggesting that the material’s persistence may offer prolonged support during nerve regeneration [77]. Currently, NeuroShield^®^ (Checkpoint Surgical, Inc. Cleveland, OH, USA) is the only commercially available chitosan-based nerve wrap, and there is a limited number of preclinical and clinical studies evaluating its efficacy [78].

### 3.7. Human Epineural Patch, a Novel Strategy for Nerve Protection

One of the novel approaches explored in our laboratory is the human Epineural Patch (hEP), a nerve protector derived from the human epineurium—the outermost layer of peripheral nerves [79]. In this study, we investigated the efficacy of the hEP in a complex rat sciatic nerve injury model that included crush injury, transection, and end-to-end repair, conditions designed to replicate clinically relevant nerve trauma. Unlike other biological wraps, which often degrade prematurely or lack sufficient structural integrity, our findings demonstrate that the hEP provides a durable protective effect supporting nerve healing without inducing an immune response [79]. Following sciatic nerve repair, the application of the hEP to the injury site enhanced nerve regeneration and vascularization, as evidenced by increased expression of vasculogenic (VEGF and vFV) and neurogenic (laminin B, NGF, and S 100) markers. These results confirm the hEP’s potential to promote nerve regeneration. Furthermore, compared to the human amnion membrane (hAM), hEP application significantly improved long-term functional outcomes, as validated by standard functional tests. Thus, the hEP presents a novel and valuable protective option for peripheral nerve reconstruction, offering an alternative tool in regenerative medicine [79].

In our previous research, we have explored the various applications of the human epineurium, including epineural jackets, sheaths, and conduits. These studies have confirmed the neuroregenerative, anti-inflammatory, and immunomodulatory potential of epineurium-based products [80,81,82,83,84,85]. Given its unique neuroregenerative properties, the hEP represents a valuable addition to the repertoire of biological nerve wraps, making it a strong candidate for clinical translation. These findings are promising; however, the clinical potential of the hEP should be further validated through clinical studies.

### 3.8. A New Perspective on Biological Nerve Protection

Nerve protectors are not limited to traditional wrap formats. New products, such as the VersaWrap (Alafair Biosciences, Inc, Austin, TX, USA), initially designed for tendon, ligament, and skeletal muscle protection, represent a bioresorbable plant-based hydrogel composed of hyaluronic acid (HA) and alginate [86]. To expand these applications, recent studies have explored its potential in enhancing nerve regeneration following PNI. VersaWrap can be implanted as either a sheet or gel via a syringe, offering an innovative approach to nerve protection [29,87]. It features a nano-porous structure that allows the passage of small molecules, including growth factors while acting as a barrier to larger proteins and fibroblasts. The hydrogel gradually resorbs within 3 to 6 months following implantation, though residual biopolymer traces may persist for up to two years, ensuring extended protection during the critical phases of nerve regeneration and healing [29]. Preliminary clinical data support its safety and efficacy in patients undergoing revision surgery for upper-extremity compressive neuropathies. Despite the study’s limitations—such as a small sample size and retrospective design—all patients experienced symptomatic improvement, expressed postoperative satisfaction, and did not require further surgical intervention within the observed postoperative period [87].

## 4. Synthetic Nerve Protectors

While biological wraps leverage nature’s regenerative mechanisms, the development of synthetic nerve protectors arose from the need to overcome the limitations of biological wraps, marking the beginning of research on synthetic products to enhance nerve regeneration [88]. In recent years, biodegradable polymers have been increasingly utilized in the fabrication of scaffolds for neural tissue engineering, with polycaprolactone (PCL) being the most extensively studied for nerve wrap applications [89]. In contrast, polymers, such as polyglycolic acid (PGA), polylactic acid (PLA), and their copolymer poly(lactic-co-glycolic) acid (PLGA), degrade into acidic byproducts, which can induce localized inflammatory responses and create a suboptimal microenvironment, ultimately impairing peripheral nerve regeneration. PCL-based wraps offer controlled degradation rates, allowing them to gradually degrade over time as the nerve undergoes recovery [90]. Furthermore, their mechanical properties can be tailored to match the physiological needs of the regenerating nerve, providing a scaffold that guides axonal outgrowth while minimizing perineural fibrosis. 

### Polycaprolactone (PCL) Wraps for Nerve Protection

Initially developed for controlled drug delivery and tissue engineering, poly-ε-caprolactone (PCL) has found a vital role in nerve repair [30]. Its biodegradable nature, along with its ability to mimic the extracellular matrix and integrate with various coatings and polymers, improves biocompatibility and mechanical strength [91]. Its widespread use is further driven by its affordability, natural resorption over several months, and nonimmunogenic properties, making it a practical choice for biomedical applications. However, despite advantages it should be noted that synthetic polymers such as PCL are inherently hydrophobic, which can impair cellular adhesion and potentially trigger inflammatory or immune responses. Recent advances in electrospinning techniques have enabled the creation of PCL scaffolds with interconnected macropores, forming nerve wraps that reduce scarring and promote healing with minimal inflammation [92]. In a study by Sarhane et al., an innovative nanofiber PCL wrap was evaluated in a rat model involving primary nerve transection followed by direct repair. At five weeks post-surgery, the PCL wrap demonstrated efficacy in enhancing neuroregeneration. The macroporous structure of the wrap facilitated controlled tissue ingrowth and preserved nerve continuity. Histological assessments revealed minimal scarring and smooth integration with surrounding tissues, with no signs of nerve damage or adverse inflammatory responses. In contrast, the control group exhibited significant fibrosis, compromising neural integrity [30]. The effectiveness of PCL can be further improved when it is coated with different materials. A study by Harley-Troxe et al. showed that PCL fibers coated with graphene oxide promote axonal growth in a rat model of sciatic nerve injury, emphasizing the promise of these composite materials in nerve regeneration applications [89]. However, clinical evidence remains limited, and further research is necessary to validate their efficacy in human applications. 

## 5. Hybrid Synthetic-Biological Wraps as the Protective Barrier

Hybrid wraps represent a bridge between biological and synthetic nerve protection options, addressing unfavorable outcomes of peripheral nerve injury, such as axonal escape and excessive scarring, which often compromise functional recovery. Currently, the only reported hybrid constructs are nanofibrous membranes composed of polycaprolactone (PCL) and amniotic membrane (AM), which have shown significant potential in addressing the neurobiology of an injury by modulating macrophage polarization and regulating the inflammatory microenvironment [93]. PCL/AM hybrid wraps are known to reduce fibrosis and preserve the regenerative pathway. PCL provides mechanical stability, while AM fosters biological integration, together creating an optimal framework for axonal regrowth and Schwann cell migration. Evidence from preclinical studies using rat sciatic nerve injury models further supports the therapeutic potential of these materials. PCL-amnion nanofibrous membranes decrease the expression of pro-inflammatory cytokines such as IL-6 and TNF-α while increasing levels of anti-inflammatory cytokines like IL-10 and IL-13, thereby promoting an M2 macrophage-dominant healing response. This immunomodulatory effect contributes to a reduction in fibrotic tissue formation by suppressing the excessive extracellular matrix deposition, ultimately preventing scar-related inhibition of axonal regrowth. [31]. At 16 weeks post-implantation, these scaffolds have been shown to enhance functional recovery by facilitating Schwann cell proliferation, accelerating axon regeneration, and reducing muscle denervation. Moreover, they contribute to higher axon numbers, increased myelin sheath thickness, and improved overall nerve maturity [94]. 

## 6. Limitations of Commercial Nerve Wraps

Although significant advancements have been made in the development of biological and synthetic wraps and materials for nerve protection and regeneration, no standardized guidelines exist for their optimal application in acute versus chronic nerve injuries. While nerve protectors serve as a barrier shielding injured nerves from surrounding tissues, they also present certain limitations (Table 4). Biocompatibility concerns, particularly with animal-derived materials, may trigger immune reactions or be unsuitable for patients due to ethical or religious considerations. Even biocompatible options can induce low-grade inflammatory responses over time, and their biodegradation may be slower than expected, potentially leading to fibrosis or foreign body reactions [95,96]. Additionally, standardized sizes may not accommodate all nerve repairs, resulting in mismatches that can cause iatrogenic constriction or slippage, thereby reducing effectiveness [11]. Proper placement of nerve wraps and protectors is crucial, as excessive tension can hinder nerve regeneration and increase the risk of scarring, fibrosis, and adhesion formation [2].

## 7. Future Perspectives

Looking ahead, the next generation of nerve protectors must move beyond the limitations of current materials. A comprehensive, multilevel intervention can substantially improve functional recovery and, consequently, the long-term quality of life of patients with peripheral nerve injuries. Future strategies may involve the integration of nerve-protecting wraps with advanced therapeutic approaches such as stem cell therapies and controlled delivery of the growth factors. Incorporation of stem cells into nerve wraps may enhance nerve regeneration leading to cell differentiation into neural lineages, including motor neurons and Schwann cells. Moreover, stem cells can promote axonal growth by secreting paracrine factors, modulating the inflammatory milieu, facilitating tissue remodeling, reducing fibrosis, and enhancing repair quality. Furthermore, the release of neurogenic and angiogenic growth factors, such as NGF, BDNF, VEGF, and PDGF, respectively, in a controlled and sustained manner will further promote axonal growth and enhance neuronal survival while mitigating trauma-induced muscle atrophy. To be effective, therapeutic constructs must be both durable and capable of adapting to the dynamic nature of nerve regeneration. Therefore, future innovative approaches should prioritize the development of personalized and/or customizable nerve wraps and protectors that would address these challenges effectively.

## 8. Conclusion

Nerve protectors, including biological and synthetic wraps, have significantly advanced the management of peripheral nerve injuries (PNIs). They serve as an adjuvant treatment option in severe cases, where creating a barrier between the repaired nerves and surrounding tissues is essential to prevent fibrosis and tissue adhesions. Depending on the type and severity of the PNI, the application of nerve protective materials should be tailored to the individual patient’s needs to achieve optimal outcomes. While nerve protectors enhance nerve regeneration, challenges such as biocompatibility and immune reactions remain. Further research is required to assess long-term neuroregenerative effects and optimize clinical applications of nerve wraps. Continued innovation and rigorous clinical evaluations are essential to ensuring their effectiveness and achieving consistent, reliable outcomes.

## Figures and Tables

**Table 1:** Comparison of nerve wrapping materials based on key characteristics.

Material	Success Rate	Biological Response	Immunogenicity	Resorption Rate	Clinical Applicability
Vein	Moderate	Promotes tissue regeneration, low scar formation	Low	N/A	Recurrent and persistent compressive neuropathies
Hypothenar Fat Flap	Moderate	Supports regeneration, reduces scarring	Low	N/A	Recurrent and persistent compressive neuropathies
Human Amniotic Membrane (hAM)	High	Strong regenerative properties, anti-inflammatory, antifibrotic, antibacterial	Low	1–3 months	Recurrent and persistent compressive neuropathies
Collagen Type I	Moderate	Supports cellular growth and collagen formation	Moderate	4–8 months 36–48 months	Recurrent and persistent compressive neuropathies
Porcine Small Intestinal Submucosa	High	Supports tissue regeneration, some risk of immune response	Moderate	3 months	Recurrent and persistent compressive neuropathies
Chitosan	Limited data	Biodegradable, supports tissue growth, low inflammation	Low	3–18 months	Experimental/ preclinical stage
Hyaluronic acid	High	Enhances tissue repair, anti-inflammatory	Low	3–6 months	Recurrent and persistent compressive neuropathies
Human Epineural Patch (hEP)	Limited data	Promotes tissue regeneration, low scar formation, low fibrosis, anti-inflammatory	Low	12 months	Experimental/ preclinical stage
Poly-ε-caprolactone (PCL)	Limited data	Biocompatible, supports cellular attachment	Moderate	18–36 months	Experimental/ preclinical stage
Poly-ε-caprolactone (PCL)/Amnion	Limited data	Anti-inflammatory, antifibrotic, anti-adhesion properties, promotion of nerve generation	Low	Limited data	Experimental/ preclinical stage

**Table 2:** FDA-approved nerve protectors.

510K Approval	Product Name	Material	Company	Address	FDA Approval Year
K041620	NeuraWrap™	Type I bovine collagen and chondroitin-6-sulfate	Integra LifeSciences, Corp.	Plainsboro, NJ, USA	2004
K060952	NeuroMend™	Type I bovine collagen and chitosan	Styker, Corp.	Kalamazoo, MI, USA	2006
K132660	AxoGuard^®^ Nerve Protector	Porcine small intestinal submucosa (SIS) extracellular collagen matrix (ECM)	Axogen, Inc.	Alachua, FL, USA	2014
K190246	NeuroShield™	Chitosan	Checkpoint Surgical, Inc.	Cleveland, OH, USA	2019
K210665	Nerve Tape^®^	Porcine small intestinal submucosa (SIS) extracellular collagen matrix (ECM)	BioCircuit Technologies, Inc.	Atlanta, GA, USA	2022
K223640	Axoguard^®^ HA+ Nerve Protector	Porcine small intestinal submucosa (SIS) extracellular collagen matrix (ECM), sodium hyaluronate, and sodium alginate	Axogen, Inc.	Alachua, FL, USA	2023
K232029	Versawrap nerve protector	Calcium alginate and hyaluronic acid	Alafair Biosciences, Inc.	Austin, TX, USA	2023

**Table 3:** First reports on the use of various materials as nerve protecting wraps.

Researcher	Year	Nerve Wrap Material	Animal
Biological Nerve Wraps			
Masear et al. [10]	1989	Vein	Rat
Strickland JW et al. [25]	1969	Hypothenar Fat Flap	Rat
O’ Neill AC et al. [26]	2009	Human Amniotic Membrane (hAM)	Rat
Kim PD et al. [27]	2010	Collagen Type I	Rat
Kokkalis ZT et al. [28]	2011	Porcine Small Intestinal Submucosa	Rat
Hones KM [29]	2023	Hyaluronic acid	Rat
Synthetic Nerve Wraps			
Sahrane et al. [30]	2019	Poly-ε-caprolactone (PCL)	Rat
Hybrid Nerve Wraps			
Liu et al. [31]	2023	Poly-ε-caprolactone (PCL)/Amnion	Rat

**Table 4:** Advantages and disadvantages of nerve wrapping materials.

Material	Advantages	Disadvantages
Vein	Cost-effective; biologically inert	Donor site morbidity; limited mechanical strength; extended surgery time
Hypothenar Fat Flap	Easy local application; well-vascularized	Donor site morbidity; extended surgery time; limited coverage
Human Amniotic Membrane (hAM)	No donor site morbidity; anti-inflammatory and non-immunogenic properties; low incidence of reported complications; relatively affordable; available in various sizes and shapes	Potential risk of disease transmission; requires specific regulatory approvals; limited mechanical strength and handling properties, rapid degradation rate
Collagen Type I	No donor site morbidity; off-the-shelf availability; reduces the risk of long-term foreign body response; selective permeability	Degradation rate can vary and might not match nerve regeneration timing; may elicit mild immune response depending on the source
Porcine Small Intestinal Submucosa	No donor site morbidity; durable; flexible; easy application; full degradation within 4 months; non-immunogenic when decellularized	Ethical/religious concerns for some patients; may elicit mild immune responses depending on processing
Chitosan	Transparency; flexibility and resistance to collapse	Variable degradation rate; low mechanical strength; limited clinical data; limited commercial availability
Hyaluronic acid	No donor site morbidity	Rapid degradation rate
Human Epineural Patch (hEP)	No donor site morbidity; neurogenic, vasculogenic, anti-inflammatory; non-immunogenic properties	Requires regulatory approval; lack of clinical data
Poly-ε-caprolactone (PCL)	Excellent mechanical properties; easy production	Poor cell affinity; poor hydrophilicity; risk of inflammation; potential for immune rejection
Poly-ε-caprolactone (PCL)/Amnion	Improved mechanical strength of AM through the incorporation of PCL nanofibers; high porosity; sustained release of exogenous growth factors	Poor cell affinity; poor hydrophilicity

## Data Availability

No new data were created or analyzed in this study. Data sharing is not applicable to this article.

## Abbreviations

The following abbreviations are used in this manuscript:

PNIs	Peripheral nerve injuries
hAM	Human amniotic membrane
PSIS	Porcine small intestinal submucosa
PCL	Poly-ε-caprolactone
VW	Vein wrapping
bFGF	Basic fibroblast growth factor
HO-1	Heme oxygenase-1
IL-4	Interleukin-4
IL-10	Interleukin-10
ARG1	Arginase-1
ADSCs	Adipose-derived stem cells
HLA	Human leukocyte antigen
ECM	Extracellular matrix
VEGF	Vascular endothelial growth factor
NGF	Nerve growth factor
FDA	Food and Drug Administration
PGA	Polyglycolic acid
PLA	Polylactic acid
PLGA	Poly(lactic-co-glycolic) acid
TNF-α	Tumor necrosis factor-alpha
hEP	Human Epineural Patch
HA	Hyaluronic acid
NGF	Nerve growth factor
BDNF	Brain-derived neurotrophic factor
VEGF	Vascular endothelial growth factor
PDGF	Platelet-derived growth factor

## References

[1] Aman M., Zimmermann K.S., Thielen M., Thomas B., Daeschler S., Boecker A.H., Stolle A., Bigdeli A.K., Kneser U., Harhaus L. (2022). An Epidemiological and Etiological Analysis of 5026 Peripheral Nerve Lesions from a European Level I Trauma Center. J. Pers. Med..

[2] Siemionow M., Brzezicki G. (2009). Current techniques and concepts in peripheral nerve repair. Int. Rev. Neurobiol..

[3] Bateman E.A., Pripotnev S., Larocerie-Salgado J., Ross D.C., Miller T.A. (2024). Assessment, management, and rehabilitation of traumatic peripheral nerve injuries for non-surgeons. Muscle Nerve.

[4] Dellon E.S., Dellon A.L. (1993). The first nerve graft, Vulpian, and the nineteenth-century neural regeneration controversy. J. Hand Surg. Am..

[5] Thomson S.E., Ng N.Y., Riehle M.O., Kingham P.J., Dahlin L.B., Wiberg M., Hart A.M. (2022). Bioengineered nerve conduits and wraps for peripheral nerve repair of the upper limb. Cochrane Database Syst. Rev..

[6] Rath S., Green C.J. (1991). Selectivity of distal reinnervation of regenerating mixed motor and sensory nerve fibers across muscle grafts in rats. Br. J. Plast. Surg..

[7] Supra R., Agrawal D.K. (2023). Peripheral nerve regeneration: Opportunities and challenges. J. Spine Res. Surg..

[8] Dy C.J., Aunins B., Brogan D.M. (2018). Barriers to epineural scarring: Role in treatment of traumatic nerve injury and chronic compressive neuropathy. J. Hand Surg. Am..

[9] Thakker A., Sharma S.C., Hussain N.M., Devani P., Lahiri A. (2021). Nerve wrapping for recurrent compression neuropathy: A systematic review. J. Plast. Reconstr. Aesthetic Surg..

[10] Masear V. Venous wrapping of nerves to prevent scarring. Proceedings of the 44th Annual Meeting of the American Society for Surgery of the Hand.

[11] Mayrhofer-Schmid M., Klemm T.T., Aman M., Kneser U., Eberlin K.R., Harhaus L., Boecker A.H. (2023). Shielding the Nerve: A Systematic Review of Nerve Wrapping to Prevent Adhesions in the Rat Sciatic Nerve Model. J. Pers. Med..

[12] Kehoe S., Zhang X.F., Boyd D. (2012). FDA-approved guidance conduits and wraps for peripheral nerve injury: A review of materials and efficacy. Injury.

[13] Fakhr M.J., Kavakebian F., Ababzadeh S., Rezapour A. (2024). Challenges and Advances in Peripheral Nerve Tissue Engineering Critical Factors Affecting Nerve Regeneration. J. Tissue Eng. Regen. Med..

[14] Taylor C.S., Haycock J.W., Phillips J.B., Hercher D., Hausner T. (2022). Biomaterials and scaffolds for repair of the peripheral nervous system. Peripheral Nerve Tissue Engineering and Regeneration.

[15] Fornasari B.E., Carta G., Gambarotta G., Raimondo S. (2020). Natural-based biomaterials for peripheral nerve injury repair. Front. Bioeng. Biotechnol..

[16] Liu X., Duan X. (2023). Mechanisms and treatments of peripheral nerve injury. Ann. Plast. Surg..

[17] Crabtree J.R., Mulenga C.M., Tran K., Feinberg K., Santerre J.P., Borschel G.H. (2024). Biohacking Nerve Repair: Novel Biomaterials, Local Drug Delivery, Electrical Stimulation, and Allografts to Aid Surgical Repair. Bioengineering.

[18] Lam T.C., Leung Y.Y. (2024). Innovations in peripheral nerve regeneration. Bioengineering.

[19] Arkansas Blue Cross Blue Shield Peripheral Nerve Repair and Reconstruction. https://journals.lww.com/jbjsjournal/abstract/2013/12040/peripheral_nerve_repair_and_reconstruction.9.aspx.

[20] Mukai M., Uchida K., Hirosawa N., Murakami K., Inoue G., Miyagi M., Shiga Y., Sekiguchi H., Inage K., Orita S. (2022). Frozen vein wrapping for chronic nerve constriction injury reduces sciatic nerve allodynia in a rat model. BMC Neurosci..

[21] Papatheodorou L.K., Sotereanos D.G., Sotereanos D.G., Papatheodorou L.K. (2020). Vein wrapping of peripheral nerves: Surgical technique. Compressive Neuropathies of the Upper Extremity.

[22] Hirosawa N., Uchida K., Kuniyoshi K., Murakami K., Inoue G., Miyagi M., Matsuura Y., Orita S., Inage K., Suzuki T. (2017). Vein wrapping facilitates basic fibroblast growth factor-induced heme oxygenase-1 expression following chronic nerve constriction injury. J. Orthop. Res..

[23] Mukai M., Uchida K., Hirosawa N., Murakami K., Kuniyoshi K., Inoue G., Miyagi M., Sekiguchi H., Shiga Y., Inage K. (2019). Wrapping With Basic Fibroblast Growth Factor-Impregnated Collagen Sheet Reduces Rat Sciatic Nerve Allodynia. J. Orthop. Res..

[24] Hirosawa N., Uchida K., Kuniyoshi K., Murakami K., Inoue G., Miyagi M., Matsuura Y., Orita S., Inage K., Suzuki T. (2018). Vein wrapping promotes M2 macrophage polarization in a rat chronic constriction injury model. J. Orthop. Res..

[25] Strickland J.W., Idler R.S., Lourie G.M., Plancher K.D. (1996). The hypothenar fat pad flap for management of recalcitrant carpal tunnel syndrome. J. Hand Surg..

[26] O’Neill A.C., Randolph M.A., Bujold K.E., Kochevar I.E., Redmond R.W., Winograd J.M. (2009). Photochemical Sealing Improves Outcome Following Peripheral Neurorrhaphy. J. Surg. Res..

[27] Kim P.D., Hayes A., Amin F., Akelina Y., Hays A.P., Rosenwasser M.P. (2010). Collagen nerve protector in rat sciatic nerve repair: A morphometric and histological analysis. Microsurgery.

[28] Kokkalis Z.T., Pu C., Small G., Weiser R.W., Venouziou A., Sotereanos D.G. (2010). Assessment of Processed Porcine Extracellular Matrix as a Protective Barrier in a Rabbit Nerve Wrap Model. J. Reconstr. Microsurg..

[29] Hones K.M., Nichols D.S., Barker H., Cox E., Hones J.A., Chim H. (2023). Outcomes following use of VersaWrap nerve protector in treatment of patients with recurrent compressive neuropathies. Front. Surg..

[30] Sarhane K.A., Ibrahim Z., Martin R., Krick K., Cashman C.R., Tuffaha S.H., Broyles J.M., Prasad N., Yao Z.-C., Cooney D.S. (2019). Macroporous nanofiber wraps promote axonal regeneration and functional recovery in nerve repair by limiting fibrosis. Acta Biomater..

[31] Liu C., Liu D., Zhang X., Hui L., Zhao L. (2023). Nanofibrous polycaprolactone/amniotic membrane facilitates peripheral nerve regeneration by promoting macrophage polarization and regulating inflammatory microenvironment. Int. Immunopharmacol..

[32] Langdell H.C., Zeng S.L., Pidgeon T.S., Mithani S.K. (2023). Recalcitrant Neuropathies in the Upper Extremity. J. Hand Surg. Glob. Online.

[33] Oh S.H., Chung J.I. (2023). Oblique axis hypothenar free flaps: Tips for harvesting larger flaps with minimal donor site morbidity. Arch. Plast. Surg..

[34] Shin A., Saffari T., Saffari S., Vyas K., Mardini S. (2022). Role of adipose tissue grafting and adipose-derived stem cells in peripheral nerve surgery. Neural Regen. Res..

[35] Danoff J.R., Lombardi J.M., Rosenwasser M.P. (2014). Use of a pedicled adipose flap as a sling for anterior subcutaneous transposition of the ulnar nerve. J. Hand Surg. Am..

[36] Dibbs R.P., Ali K., Sarrami S.M., Koshy J.C. (2021). Revision peripheral nerve surgery of the upper extremity. Semin. Plast. Surg..

[37] Kingham P.J., Kalbermatten D.F., Mahay D., Armstrong S.J., Wiberg M., Terenghi G. (2007). Adipose-derived stem cells differentiate into a Schwann cell phenotype and promote neurite outgrowth in vitro. Exp. Neurol..

[38] Ching R.C., Wiberg M., Kingham P.J. (2018). Schwann cell-like differentiated adipose stem cells promote neurite outgrowth via secreted exosomes and RNA transfer. Stem Cell Res. Ther..

[39] Riccio M., Gravina P., Pangrazi P.P., Cecconato V., Gigante A., De Francesco F. (2023). Ulnar nerve anteposition with adipofascial flap, an alternative treatment for severe cubital syndrome. BMC Surg..

[40] Mamede A.M.A., Botelho A.C. (2015). Amniotic Membrane: Origin, Characterization, and Medical Applications.

[41] Wolfe E.M., Mathis S.A., Muñoz N.d.l.O., Ovadia S.A., Panthaki Z.J. (2022). Comparison of human amniotic membrane and collagen nerve wraps around sciatic nerve reverse autografts in a rat model. Biomater. Biosyst..

[42] Leal-Marin S., Kern T., Hofmann N., Pogozhykh O., Framme C., Börgel M., Figueiredo C., Glasmacher B., Gryshkov O. (2020). Human Amniotic Membrane: A review on tissue engineering, application, and storage. J. Biomed. Mater. Res. Part B Appl. Biomater..

[43] McClendon D.C., Su J., Smith D.W. (2023). Human amniotic allograft in hand surgery. J. Hand Surg. Am..

[44] Yu L.-M., Yu C.-Y., Zhang Z.-Y., Yang J., Fan Z.-H., Wang D.-L., Wang Y.-Y., Zhang T. (2019). Fresh human amniotic membrane effectively promotes the repair of injured common peroneal nerve. Neural Regen. Res..

[45] Mirzayan R., Russo F., Yang S.-J.T., Lowe N., Shean C.J., Harness N.G. (2022). Human Amniotic Membrane Wrapping of the Ulnar Nerve During Cubital Tunnel Surgery Reduces Recurrence of Symptoms. Arch. Bone Jt. Surg..

[46] Fénelon M., Catros S., Meyer C., Fricain J.-C., Obert L., Auber F., Louvrier A., Gindraux F. (2021). Applications of Human Amniotic Membrane for Tissue Engineering. Membranes.

[47] Li X., Zhang X., Hao M., Wang D., Jiang Z., Sun L., Gao Y., Jin Y., Lei P., Zhuo Y. (2022). The application of collagen in the repair of peripheral nerve defect. Front. Bioeng. Biotechnol..

[48] Jiang M., Chen M., Liu N. (2024). Interactions between Schwann cell and extracellular matrix in peripheral nerve regeneration. Front. Neurol..

[49] Hardin-Young J., Carr R.M., Downing G.J., Condon K.D., Termin P.L. (2000). Modification of native collagen reduces antigenicity but preserves cell compatibility. Biotechnol. Bioeng..

[50] Eleftheriadou D., Phillips J.B., Phillips J.B., Hercher D., Hausner T. (2022). Collagen biomaterials for nerve tissue engineering. Peripheral Nerve Tissue Engineering and Regeneration.

[51] Abedi M., Shafiee M., Afshari F., Mohammadi H., Ghasemi Y. (2023). Collagen-Based Medical Devices for Regenerative Medicine and Tissue Engineering. Appl. Biochem. Biotechnol..

[52] Chen S., Gao Y.-B., Liu Z.-G., Lin G.-D., Guo Y., Chen L., Huang B.-T., Yin Y.-B., Yang C., Sun L.-Y. (2021). Safety and efficacy of a nerve matrix membrane as a collagen nerve wrapping: A randomized, single-blind, multicenter clinical trial. Neural Regen. Res..

[53] Wong G.C., Chung K.C. (2024). Bioengineered nerve conduits and wraps. Hand Clin..

[54] Spielman A.F., Sankaranarayanan S., Skowronski P., Lessard A.-S., Panthaki Z. (2020). Recurrent and persistent carpal tunnel syndrome: “Triple-therapy approach”. J. Orthop..

[55] Integra LifeSciences Corporation NeuraWrap™ Nerve Protector. https://www.integranerve.com/nerve-protector.

[56] Stryker Corporation NeuroMend™ Wrap. https://www.stryker.com/us/en/trauma-and-extremities/products/neuromend.html.

[57] Downey M.S. (2015). A guide to nerve wrapping for tarsal tunnel surgery. Podiatry Today.

[58] Rault I., Frei V., Herbage D., Abdul-Malak N., Huc A. (1996). Evaluation of different chemical methods for cros-linking collagen gel, films and sponges. J. Mater. Sci. Mater. Med..

[59] Mariani E., Lisignoli G., Borzì R.M., Pulsatelli L. (2019). Biomaterials: Foreign Bodies or Tuners for the Immune Response?. Int. J. Mol. Sci..

[60] Hanwright P.J., Rath J.B., von Guionneau N., Slavin B., Pinni S., Zlotolow D., Shores J., Dellon A.L., Tuffaha S.H. (2021). The Effects of a Porcine Extracellular Matrix Nerve Wrap as an Adjunct to Primary Epineurial Repair. J. Hand Surg..

[61] Yi J.-S., Lee H.-J., Lee H.-J., Lee I.-W., Yang J.-H. (2013). Rat Peripheral Nerve Regeneration Using Nerve Guidance Channel by Porcine Small Intestinal Submucosa. J. Korean Neurosurg. Soc..

[62] Li T., Javed R., Ao Q. (2021). Xenogeneic decellularized extracellular matrix-based biomaterials for peripheral nerve repair and regeneration. Curr. Neuropharmacol..

[63] Grandizio L.C., Maschke S., Evans P.J. (2018). The management of persistent and recurrent cubital tunnel syndrome. J. Hand Surg. Am..

[64] Imran R., George S., Jose R., Shirley C., Power D.M. (2022). Clinical outcomes following neurolysis and porcine collagen extracellular matrix wrapping of scarred nerves in revision carpal tunnel decompression. J. Plast. Reconstr. Aesthetic Surg..

[65] Fones L., DePascal M., Ilyas A.M. (2024). Use of nerve wraps in the upper extremity. SurgiColl.

[66] Burahee A.S., Duraku L.S., Bosman R., Shirley C., van der Oest M.J., Zuidam M.J., Power D.M. (2024). Porcine submucosal extracellular matrix wrapping of the ulnar nerve in revision cubital tunnel surgery. J. Plast. Reconstr. Aesthetic Surg..

[67] Fujii M., Tanaka R. (2022). Porcine Small Intestinal Submucosa Alters the Biochemical Properties of Wound Healing: A Narrative Review. Biomedicines.

[68] Jordaan P., Uhiara O., Power D. (2019). Management of the scarred nerve using porcine submucosa extracellular matrix nerve wraps. J. Musculoskelet. Surg. Res..

[69] Eberlin K.R., Safa B., Buntic R., Rekant M.S., Richard M.J., Styron J.F., Bendale G., Isaacs J. (2024). Usability of Nerve Tape: A Novel Sutureless Nerve Coaptation Device. J. Hand Surg..

[70] Bendale G.S., Sonntag M., Clements I.P., Isaacs J.E. (2022). Biomechanical Testing of a Novel Device for Sutureless Nerve Repair. Tissue Eng. Part C Methods.

[71] Zeng W., Osterman M., Stern P.J. (2019). Inflammatory reactions to xenogenic nerve wraps: A report of three cases. JBJS Case Connect..

[72] Zhang M., An H., Zhang F., Jiang H., Wan T., Wen Y., Han N., Zhang P. (2023). Prospects of Using Chitosan-Based Biopolymers in the Treatment of Peripheral Nerve Injuries. Int. J. Mol. Sci..

[73] Yao P., Li P., Jiang J.J., Li H.Y. (2018). Anastomotic stoma coated with chitosan film as a betamethasone dipropionate carrier for peripheral nerve regeneration. Neural Regen. Res..

[74] Bąk M., Gutlowska O.N., Wagner E., Gosk J. (2017). The role of chitin and chitosan in peripheral nerve reconstruction. Polym. Med..

[75] Kołodziejska M., Jankowska K., Klak M., Wszoła M. (2021). Chitosan as an Underrated Polymer in Modern Tissue Engineering. Nanomaterials.

[76] Ke C.-L., Deng F.-S., Chuang C.-Y., Lin C.-H. (2021). Antimicrobial Actions and Applications of Chitosan. Polymers.

[77] Aranaz I., Alcántara A.R., Civera M.C., Arias C., Elorza B., Caballero A.H., Acosta N. (2021). Chitosan: An Overview of Its Properties and Applications. Polymers.

[78] Jain N., Murchinson M., Rounds A., Bourland B. (2023). Hemoclip migration after revision carpal tunnel release: A case report. J. Surg. Case Rep..

[79] Siemionow M., Radecka W., Kozlowska K., Chambily L., Brodowska S., Kuc D., Filipek G., Budzynska K. (2025). Protective Effect of the Human Epineural Patch Application after Sciatic Nerve Crush Injury Followed by Nerve Transection and End-to-End Repair. Arch. Immunol. Ther. Exp..

[80] Siemionow M., Uygur S., Madajka M. (2017). Application of epineural sheath as a novel approach for fat volume maintenance. Ann Plast Surg..

[81] Siemionow M., Cwykiel J., Uygur S., Kwiecien G., Oztürk C., Szopinski J., Madajka M. (2018). Application of epineural sheath conduit for restoration of 6-cm long nerve defects in a sheep median nerve model. Microsurgery.

[82] Siemionow M., Demir Y., Mukherjee A.L. (2010). Repair of peripheral nerve defects with epineural sheath grafts. Ann. Plast. Surg..

[83] Siemionow M., Duggan W., Brzezicki G., Klimczak A., Grykien C., Gatherwright J., Nair D. (2011). Peripheral Nerve Defect Repair With Epineural Tubes Supported With Bone Marrow Stromal Cells: A preliminary report. Ann. Plast. Surg..

[84] Siemionow M., Strojny M.M., Kozlowska K., Brodowska S., Grau-Kazmierczak W., Cwykiel J. (2021). Application of Human Epineural Conduit Supported with Human Mesenchymal Stem Cells as a Novel Therapy for Enhancement of Nerve Gap Regeneration. Stem Cell Rev. Rep..

[85] Siemionow M., Tetik C., Ozer K., Ayhan S., Siemionow K., Browne E. (2002). Epineural sleeve neurorrhaphy: Surgical technique and functional results—A preliminary report. Ann. Plast. Surg..

[86] Wan R., Zhao G., Adam E.A., Selim O.A., Sarcon A.K., Reisdorf R.L., Meves A., Zhao C., Moran S.L. (2024). Evaluating the Effectiveness of Commercially Available Antiadhesion Tendon Protector Sheets in Tendon Repair Surgery Versus Tendon Repair Surgery Alone: A Preclinical Model Study. J. Hand Surg..

[87] Adu Y., Harder J., Cox C., Baum G., Hernandez E.J., MacKay B.J. (2024). Evaluating the effect of VersaWrap tendon protector on functional outcomes in operative tendon repairs. Front. Surg..

[88] Zhang M., Li C., Zhou L.P., Pi W., Zhang P.X. (2021). Polymer scaffolds for biomedical applications in peripheral nerve reconstruction. Molecules.

[89] Harley-Troxell M.E., Steiner R., Newby S.D., Bow A.J., Masi T.J., Millis N., Matavosian A.A., Crouch D., Stephenson S., Anderson D.E. (2024). Electrospun PCL Nerve Wrap Coated with Graphene Oxide Supports Axonal Growth in a Rat Sciatic Nerve Injury Model. Pharmaceutics.

[90] Jiang Y., Tang X., Li T., Ling J., Yang Y. (2022). The success of biomaterial-based tissue engineering strategies for peripheral nerve regeneration. Front. Bioeng. Biotechnol..

[91] Dias J.R., Sousa A., Augusto A., Bártolo P.J., Granja P.L. (2022). Electrospun Polycaprolactone (PCL) Degradation: An In Vitro and In Vivo Study. Polymers.

[92] Lopez J., Xin K., Quan A., Xiang S., Barone A.A.L., Budihardjo J., Musavi L., Mulla S., Redett R., Martin R. (2019). Poly(ε-Caprolactone) Nanofiber Wrap Improves Nerve Regeneration and Functional Outcomes after Delayed Nerve Repair. Plast. Reconstr. Surg..

[93] Bai J., Liu C., Kong L., Tian S., Yu K., Tian D. (2022). Electrospun Polycaprolactone (PCL)-Amnion Nanofibrous Membrane Promotes Nerve Regeneration and Prevents Fibrosis in a Rat Sciatic Nerve Transection Model. Front. Surg..

[94] Dong R., Tian S., Bai J., Yu K., Liu C., Liu L., Tian D. (2022). Electrospun Polycaprolactone (PCL)-Amnion Nanofibrous Membrane Promotes Nerve Repair after Neurolysis. J. Biomater. Appl..

[95] Nicolas C.F., Corvi J.J., Zheng Y., Park K.H., Akelina Y., Engemann A., Strauch R.J. (2022). Resorbable Nerve Wraps: Can They Be Overtightened?. J. Reconstr. Microsurg..

[96] Koenig Z.A., Burns J.C., Hayes J.D. (2022). Necrotic granulomatous inflammation after use of small intestine submucosa matrix for recurrent compression neuropathy. Plast. Reconstr. Surg. Glob. Open..

